# Induction of intestinal stemness and tumorigenicity by aberrant internalization of commensal non-pathogenic *E. coli*

**DOI:** 10.1038/cddis.2017.27

**Published:** 2017-03-16

**Authors:** Upasana Sahu, Arnab Choudhury, Suhel Parvez, Subhrajit Biswas, Sudeshna Kar

**Affiliations:** 1 Jamia Hamdard-Institute of Molecular Medicine, Jamia Hamdard, New Delhi 110062, India; 2 Department of Medical Elementology and Toxicology, Jamia Hamdard, New Delhi 110062, India; 3 Institute of Liver and Biliary Sciences, New Delhi 110070, India

## Abstract

Commensal *Escherichia coli* has been identified as a major protagonist of microbe-induced colorectal oncogenesis. Its tumour-promoting attribute is linked to the expression of DNA-damaging genotoxins. Using a constitutively invasive variant of non-pathogenic *E. coli*, we demonstrate that chronic presence of internalized *E. coli* leads to enhanced oncogenicity in colon cancer cells. Instead of genomic damage, the tumorigenic effect is mediated through an expansion of the cancer stem cell (CSC) population, likely through dedifferentiation of lineage-committed intestinal epithelial cells. Stemness-linked intestinal tumorigenicity is directly correlated to absence of microbial virulence factor expression and is specific for intestinal cells. The enriched CSC fraction remains stable in the absence of the instigating bacteria and can foster stemness traits in unexposed cells through secreted factors. Mechanistically, aberrant host invasion leads to realignment of multiple host signal transduction cascades, notably mutually re-enforcing NF-*κ*B and *β*-catenin activation, through reciprocal modulation of microbe sensing pathways Nod1/Rip2 and TLR/MyD88. The expanded tumorigenic CSC population is marked by enhanced malignancy traits, long-term self-renewal capacity and robust tumorigenic ability, both *in vitro* and *in vivo*. Our study shows that microbe-induced oncogenicity is not a strict correlate of commensal virulence and can be invoked by even non-pathogenic *E. coli* by engendering tumorigenic stemness in host cells.

*Escherichia coli* is considered as one of the principal microbial protagonist of colorectal cancer (CRC) oncogenesis, based on its extremely high prevalence in CRC tissues^1, 2^ and its role in tumorigenesis in animal models.^3^ Although of disparate genotypes, *E. coli* strains associated with CRC tissues are distinguished by their ability to attach and/or invade host intestinal epithelial cells (IECs). Neoplastic initiation and/or progression is perpetrated through host DNA damage and genomic instability by means of genotoxins like colibactin.^4, 5, 6^ However, little is known about the consequences of aberrant host-microbe interaction related to non-virulent commensal *E. coli* that lack potent genotoxic factors. It is known that luminal *E. coli* can invade IECs^7^ and there is very little difference in pro-inflammatory and pro-neoplastic signalling induced by commensal and pathogenic *E. coli*.^8^ *E. coli* has one of the strongest co-occurrence profiles in paired adenoma samples but not in paired carcinoma samples.^9^ Only a fraction of the *E. coli* species in chronically inflamed, pre-cancerous lesions harbours virulence-related genes^10^ and the proportion of tumour-associated *E. coli* with or without genotoxic islands are roughly similar in TNM stage 1, but not in advanced stage III/IV, CRC tissues,^11^ raising the prospect of benign commensal *E. coli* playing a critical role in the early events of CRC oncogenesis.

We have previously created a gain-of-function mutant form of *E. coli* K-12 (SK3842) which, through nucleoid remodelling-driven changes in its transcription profile,^12, 13^ resulted in the conversion of a traditionally extra-cellular bacteria to a constitutively invasive variant. Following host cell invasion, SK3842 establishes a protective niche for itself while hindering host cell death by manipulating expression of host proteins.^14^ Since (i) bacteria involved in provoking disease states subvert host response pathways for their survival and (ii) dysregulation of cell proliferation and apoptosis cycles is linked to tumorigenesis, we hypothesized that aberrant invasion of IECs by a non-virulent *E. coli* can elicit pro-neoplastic cellular changes.

## Results

### Multiple SK3842 infections impart cytoprotective effects to host cells

**Figure 1:**
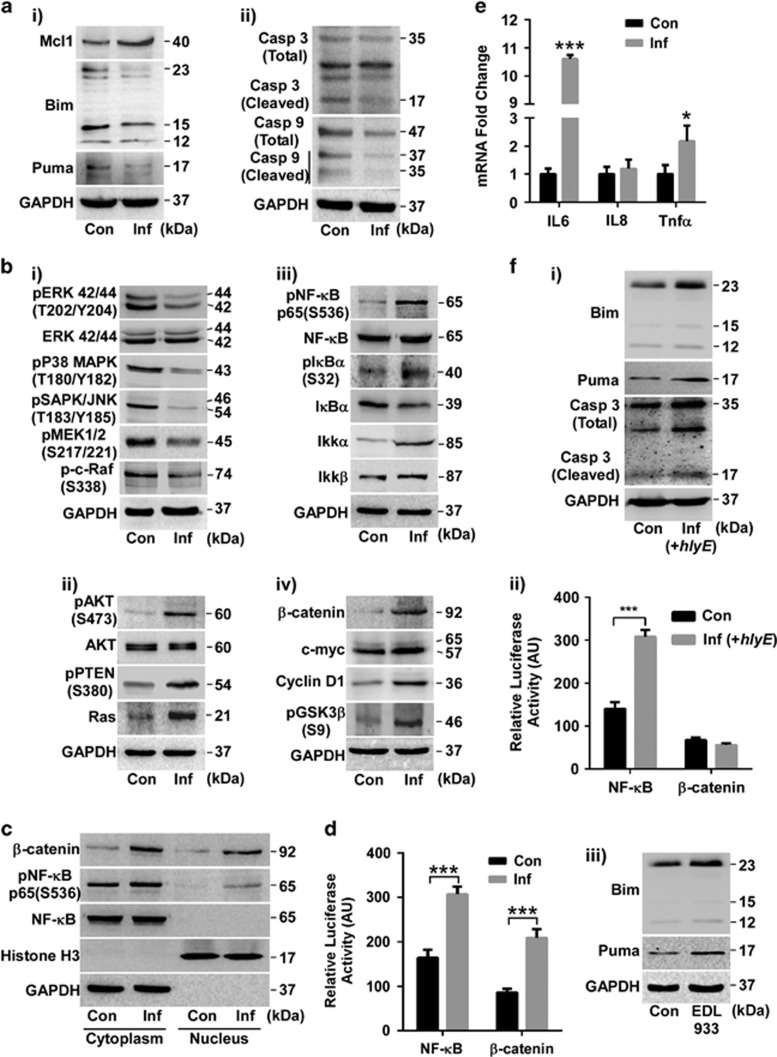
Extended presence of internalized SK3842 alters major host cell signalling. (**a**) Levels of survival-related proteins: (i) Mcl1, Bim, Puma and (ii) Caspase 3 and Caspase 9. (**b**) Changes in indicated proteins of major signal transduction modules: (i) MAPK, (ii) AKT, (iii) NF-*κ*B and (iv) *β*-catenin. (**c**) NF-*κ*B and *β*-catenin levels in cytoplasmic and nuclear fractions. (**d**) Luciferase reporter assay of NF-*κ*B and *β*-catenin transcription activity. (**e**) mRNA expression of inflammatory cytokines. (**f**) Effect of bacterial virulence factors on expression of survival-related host proteins in (i–ii) haemolysin-overexpressing SK3842 and (iii) invasive variant of O157:H7 strain. (i) Expression of Bim, Puma and activated caspase 3; (ii) Luciferase reporter assay of NF-*κ*B and *β*-catenin; (iii) Expression of Bim and Puma (after one infection). Con=control Caco-2, Inf=SK3842-infected Caco-2. Data=Mean±S.D. (three experiments); *P<0.05, **P<0.01 and ***P<0.001 *versus* control

To mimic a persistent infection milieu, we used non-differentiated epithelial colon carcinoma cell line Caco-2 and repeated infection cycles of SK3842. Multiple infection rounds resulted in increase of anti-apoptotic Mcl1, concurrent with diminished levels of pro-apoptotic Bim and Puma (Figure 1a) – the marker proteins which were correlated with enhanced cytoprotective effects during a single infection.^14^ Simultaneously, cleavage of Caspase 3 and Caspase 9 was also attenuated, confirming the cytoprotective effects of internalized SK3842.

### Extended presence of internalized *E. coli* induces major changes in host signal transduction pathways

The mitogen-activated protein kinase (MAPK) proteins – p44/42 MAPK (ERK1/2), pSAPK/JNK and p-p38 MAPK – as well as the upstream activator kinases of ERK1/2, p-c-Raf and pMEK1/2 – were all downregulated (Figure 1b(i)) in infected cells. However, PI3K/AKT pathway was significantly activated, as shown by the increased level of pAKT, and the inactive form of principal antagonist of this pathway, pPTEN (Figure 1b(ii)). Upregulation of Ras, a master regulator of both ERK and AKT pathways, indicated the repression of Ras/Raf/MEK/ERK and other MAPK pathways with simultaneous stimulation of Ras/PI3K/PTEN/AKT pathway. Activation of NF-*κ*B signalling pathway was shown by phosphorylation-driven activation of the RelA/p65 subunit of NF-*κ*B dimer, inactivation of I*κ*B*α* inhibitor and activation of IKK*α* (Figure 1b(iii)). *β*-catenin, the key transducer of the Wnt/*β*-catenin pathway, as well as two of its major downstream targets – c-myc and Cyclin D1 – were upregulated in infected cells (Figure 1b(iv)). Inactive phosphorylated form of GSK-3*β*, an important negative regulator of *β*-catenin pathway, was also increased. Since, NF-*κ*B and *β*-catenin signalling cascades play pivotal roles in CRC tumorigenesis and single SK3842 infection did not affect the status of these two pathways (Supplementary Figure S1), it proved that multiple infections are necessary to influence cell signalling networks pertinent to IEC homeostasis. Substantial accretion of both pNF-*κ*B and *β*-catenin in the nuclear fractions of infected cells confirmed the stabilization and activation of these pathways (Figure 1c). Furthermore, luciferase reporter gene assay illustrated significant enhancement of NF-*κ*B and *β*-catenin transcriptional activity (Figure 1d). NF-*κ*B activation was also verified by the upregulation of inflammatory mediators, IL6 and Tnf*α* (but not IL8), in infected cells (Figure 1e).

### Absence of bacterial virulence factor expression is necessary for host cell survival

To evaluate the influence of cryptic virulence factors on the cytoprotective effect of internalized *E. coli*, we used Hemolysin E (HlyE)-overexpressing SK3842 strain for infection. HlyE overexpression caused Bim and Puma activation and Caspase 3 cleavage (Figure 1f(i)) coupled with NF-*κ*B activation but no *β*-catenin accumulation (Figure 1f(i)), showing that expression of pathogenic factors by internalized *E. coli* is detrimental for host cells. We also used a pathogenic *E. coli* O157:H7 strain carrying the mutant HU*α* gene and studied its effect on host cells under same experimental conditions. Invasive variant of pathogenic *E. coli* caused a significant increase in Bim and Puma after just one round of infection (Figure 1f(iii)) and exacerbated cell death. Thus, cytoprotection of host cells is associated with absence of virulence factor expression from internalized *E. coli*.

### SK3842-infected cells exhibit enhanced survival and migratory characteristics

**Figure 2:**
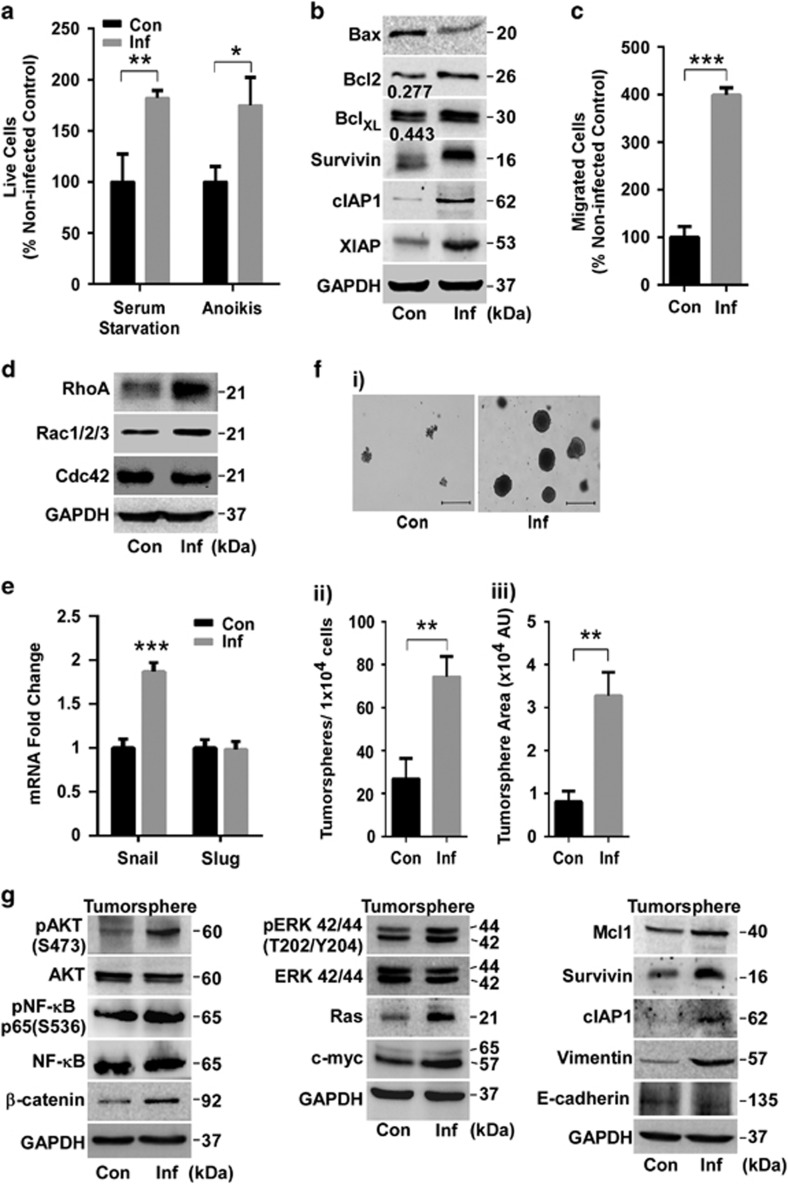
Enhanced survival, migratory and tumorigenic ability in infected host cells. (**a**) Viability of cells exposed to serum starvation and anoikis conditions. (**b**) Levels of key pro- and anti-apoptotic proteins. Numbers denote Bax/Bcl2 and Bax/Bcl_XL_ ratios. (**c**) Cell migration in Transwell assay. (**d**) Levels of Rac and Rho GTPase family proteins. (**e**) mRNA levels of epithelial-mesenchymal transcription (EMT) factors. (**f**) Tumour forming ability in soft agar colony assay, showing (i) morphology, (ii) quantitative number and (iii) arbitrary size of colonies. Scale bar=500 *μ*m. (**g**) Protein levels of key signal transduction, survival and EMT proteins in soft agar tumorspheres. Histogram=Mean±S.D. (three experiments); *P<0.05, **P<0.01 and ***P<0.001 *versus* control. AU, arbitrary units

Infected cells showed superior resistance to apoptotic cues of serum starvation and anoikis (programmed cell death provoked by loss of matrix attachment) (Figure 2a). Bax/Bcl-2 and Bax/Bcl_XL_ ratios, which act as rheostatic switches to determine the decision between survival and death, were both reduced significantly (Figure 2b). Additionally, members of inhibitor of apoptosis (IAP) family of survival proteins – Survivin, XIAP and c-IAP1 – were also upregulated. Infected cells exhibited enhanced mobility to migrate across Transwell membranes (Figure 2c). Rac1/2/3 and RhoA proteins, which regulate cell polarity, adhesion and migration, showed increased expression but no change was seen for Cdc42 (Figure 2d). EMT transcription factor Snail, but not Slug, showed significant increase (Figure 2e). However, there was no detectable change in the EMT marker proteins E-cadherin and Vimentin (data not shown), indicating a partial EMT status. These results show that invasion by SK3842 endows enhanced survival and mobility traits to IECs.

### Presence of internalized *E. coli* augments the tumorigenic potential of host cells

Anchorage-independent colony formation, a surrogate test for *in vivo* tumour malignancy, showed that infected cells developed larger, denser and more numerous colonies (Figures 2f(i–iii)). Most of the signalling pathway proteins retained their altered status in soft-agar tumorspheres, except ERK1/2 which showed activation (Figure 2g). Vimentin showed considerable increase while E-cadherin was diminished slightly but noticeably, indicating a more prominent EMT status in SK3842-infected tumorspheres (Figure 2g). Thus, long-term harbouring of SK3842 impels host IECs towards neoplastic progression through oncogenic cell signalling.

### Enhanced tumorigenicity is driven through enrichment of cancer stem cell population

**Figure 3:**
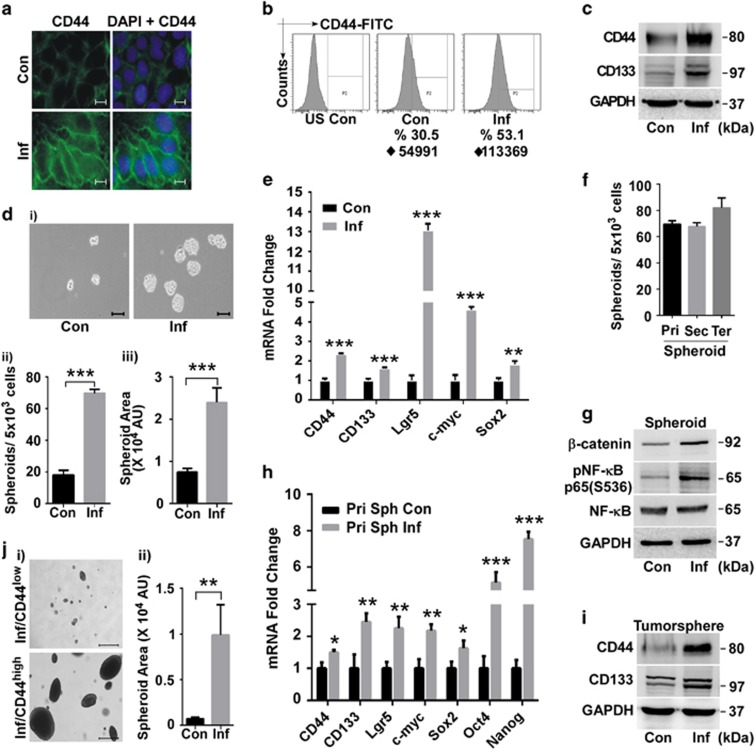
Induction of tumorigenic stem cell-like properties in infected host cell. (**a**) Immunofluorescent staining of stem cell (SC) marker CD44. Scale bar=2 *μ*m. (**b**) FACS analysis of CD44 expression. ♦=iMFI. (**c**) Protein levels of SC markers CD44 and CD133. (**d**) Sphere forming ability, showing (i) morphology, (ii) quantitative number and (iii) arbitrary size of spheroids. Scale bar=100 *μ*m. (**e**) mRNA levels of multipotency transcription factors and SC markers in adherent cells. (**f**) Self-renewing ability of microbe-induced SCs as measured by primary, secondary and tertiary spheroid formation. (**g**) Protein levels of pNF-*κ*B and *β*-catenin in spheroids. (**h**) mRNA levels of multipotency transcription factors and SC markers in spheroids. (**i**) Protein levels of SC markers in tumorspheres. (**j**) Tumorigenic ability of CD44^high^ and CD44^low^ cells of infected culture, showing (i) morphology and (ii) quantitative size. Scale bar=500 *μ*m. Data=Mean±S.D. (**d**,**e**,**h**) and Mean±S.E.M. (**j**) for three experiments; *P<0.05, **P<0.01 and ***P<0.001 *versus* control. US, Unstained; iMFI (♦)=integrated Mean Fluorescence Intensity

*β*-catenin controls the intestinal stem cell (SC) population^15^ and high *β*-catenin activity is a functional indicator of cancer stem cells (CSC).^16^ NF-*κ*B also orchestrates self-renewal signalling in SCs.^17^ Immununofluorescence microscopy showed that CD44, a downstream target of *β*-catenin and a major CSC marker, was greatly induced in infected cells (Figure 3a). Fluorescence-activated cell sorting (FACS) confirmed an increase in both the percentage of CD44^high^ cells and mean fluorescence intensity (Figure 3b). Protein levels of both CD44 and CD133, another CSC marker, were significantly elevated in infected cells (Figure 3c). Infection resulted in greatly improved SC-promoted spheroidogenic ability (Figure 3d(i)), related to spheroid number (Figure 3d(ii)) and size (Figure 3d(iii)). Apart from CD44 and CD133, intestinal SC marker Lgr5 and two major stemness-related transcription factors, Sox2 and c-myc, were also upregulated (Figure 3e). The self-renewing capacity of infected cells, as determined by their secondary and tertiary spheroid formation, was maintained over several generations (Figure 3f), indicating the expansion and subsequent stable retention of the SC fraction. pNF-*κ*B and *β*-catenin remained elevated in spheroids derived from infected cells (Figure 3g), indicating their role in maintaining stemness. All four transcription factors characteristic of multipotent SCs – Sox2, c-myc, Oct4 and Nanog – and SC markers CD44, CD133 and Lgr5 were highly upregulated in spheroid cells (Figure 3h). Soft agar tumorspheres from infected cells retained the enriched SC population (Figure 3i). FACS-sorted CD44^high^ fraction from infected culture produced large and highly dense tumorspheres in just 14 days (*versus* 21 days for adherent cells) while the CD44^low^ cells formed sparse and inconspicuous colonies (Figures 3j(i–ii)), confirming the tumorigenic capacity of the induced SCs. Collectively, anchorage-independent growth, self-renewal ability and expression of stemness markers and multipotency factors proved that tumour-promoting CSC subpopulation is expanded in SK3842-infected cells.

### Only differentiated intestinal cells are responsive towards SK3842-mediated induction of stemness

**Figure 4:**
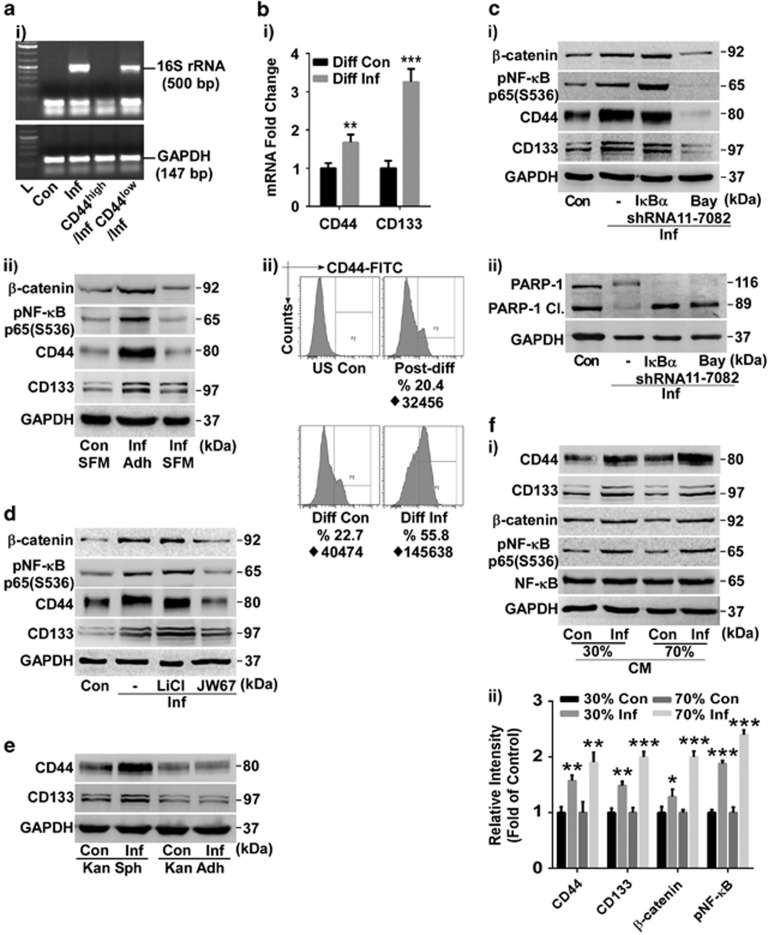
Acquisition of stemness traits through concerted action of activated NF-*κ*B and *β*-catenin. (**a**) Effect of SK3842 infection on undifferentiated cells: (i) Presence of 16S rRNA DNA in CD44^high^ and CD44^low^ cells after SK3842 re-infection. (ii) Changes in levels of indicated proteins in primary spheroids of Caco-2 cells following multiple SK3842 infections in SFM (adh=adherent). (**b**) Effect of SK3842 infection on differentiated cells: (i) mRNA expression of SC markers and (ii) Percentage of CD44^high^ cells in differentiated Caco-2 cells following multiple SK3842 infections (post-diff=starting population). (**c**) Effect of NF-*κ*B activation (I*κ*B*α* shRNA) and inhibition (Bay11-7082) on (i) expression of SC markers and (ii) PARP-1 cleavage (5 days after final infection). (**d**) Effect of *β*-catenin activation (LiCl) and inhibition (JW67) on expression of SC markers. (**e**) Expression of SC markers in bacteria-cleared infected cells under spheroid-forming and adherent conditions. (**f**) Stemness-associated protein expression in uninfected cells treated with 30 and 70% sterile conditioned media from infected cell culture: (i) Protein levels in representative blot. (ii) Summary histogram of protein levels from independent blots. Data=Mean±S.D. from three experiments (*P<0.05, **P<0.01 and ***P<0.001)

**Figure 5:**
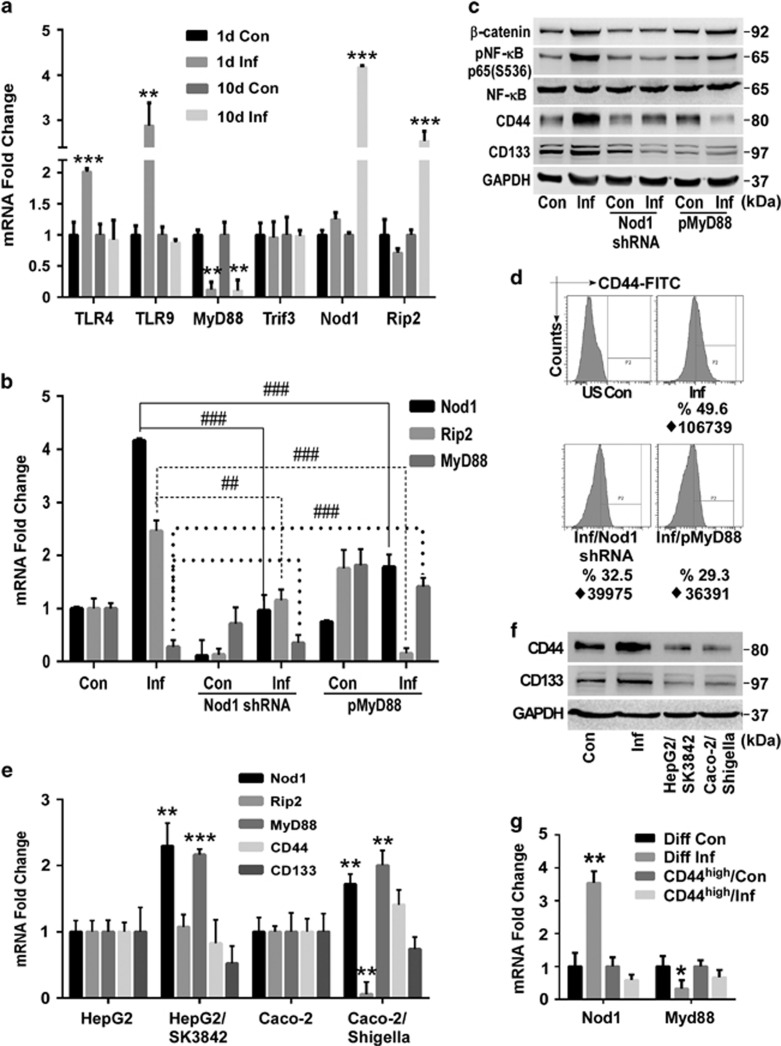
Concurrent activation of Nod1/Rip2 and suppression of TLR/MyD88 signalling induces stemness properties. (**a**) mRNA levels of components of major Pattern Recognition Receptor signalling pathways after one and three infections. (**b**) Effect of Nod1 ablation and ectopic MyD88 expression on Nod1, Rip2 and MyD88 mRNA levels. (**c**) Protein levels of pNF-*κ*B, *β*-catenin and SC markers in Nod1 knockdown and MyD88 expressing cells. (**d**) Percentage of CD44^high^ cells in Nod1 knockdown and MyD88 expressing infected cells. (**e**) Nod1, Rip2 and MyD88 mRNA expression in SK3842 infected HepG2 and *Shigella* infected Caco-2 cells. (**f**) Protein levels of SC markers in HepG2/SK3842 and Caco-2/*Shigella* cells. (**g**) mRNA levels of Nod1 and MyD88 in differentiated Caco-2 and CD44^high^ cells from pre-infected culture, following SK3842 infection. (**a–d**)=Experiments done in Caco-2 cells. Fold change=Mean±S.D. (three experiments); **P<0.01 and ***P<0.001 *versus* control, ^##^*P*<0.01 and ^###^*P*<0.001 *versus* only SK3842-infected cells

The amplified CSC population could potentially have been engendered through either expansion of existing SC population or dedifferentiation of non-stem cells. CD44^high^ and CD44^low^ cells from kanamycin-treated infected culture were exposed to an additional round of SK3842 infection in spheroid forming media (SFM). Bacterial DNA was detected only in CD44^low^ cells (Figure 4a(i)). To exclude the possibility that only microbe-induced CSCs were resistant to SK3842, we used primary spheroids of Caco-2 for infection. Neither SC markers nor pNF-*κ*B and *β*-catenin showed any noteworthy increase in infected host cells under stemness enriching conditions (Figure 4a(ii)), showing that SK3842 has limited or no ability to invade SCs. In contrast, infection of differentiated Caco-2 cells (Supplementary Figure S2) led to a significant increase of SC markers (Figure 4b(i)). FACS analysis also confirmed the amplification of CD44^high^ SC fraction in infected cells (Figure 4b(ii)). This indicated that SK3842-induced expansion of CSCs possibly occurs through dedifferentiation of non-stem IECs (Also Figure 5g).

### Concerted activation of NF-*κ*B and *β*-catenin promotes tumorigenic stemness in host cells

Since ablation of NF-*κ*B expression by p65 shRNA was lethal for infected cells we used I*κ*B*α* shRNA and suboptimal dose of Bay11-7082 to activate and diminish NF-*κ*B activity, respectively (Supplementary Figure S3A). Knockdown of I*κ*B*α* caused an accretion of not only pNF-*κ*B but also *β*-catenin (Figure 4c(i)), without any significant changes in CD44 and CD133 levels. Bay11-7082 treatment diminished *β*-catenin and SC marker levels (Figure 4c(i)). Surprisingly, not only NF-*κ*B abrogation but also its over-expression led to PARP-1 cleavage in infected cells when cultured for an extended time (Figure 4c(ii)), showing that controlled activation of NF-*κ*B is essential for survival of infected cells. Next, we used lithium chloride and JW67 to activate and inhibit *β*-catenin signalling, respectively (Supplementary Figure S3A). Lithium treatment elevated *β*-catenin, pNF-*κ*B as well as both SC markers levels slightly (Figure 4d). JW67 caused a decline in *β*-catenin, pNF-*κ*B and CD44 levels. Thus, both NF-*κ*B and *β*-catenin pathways can reciprocally boost each other but, unlike NF-*κ*B, ancillary amplification of *β*-catenin signalling can further enhance the stemness potential of infected cells (Supplementary Figure S3B).

### Continued presence of bacteria is not required for maintenance of non-differentiated state

Clearance of internalized bacteria did not diminish CD44 or CD133 levels in infected cells grown in SFM but caused a loss of stemness attributes in monolayer culture (Figure 4e), showing that the maintenance of expanded CSC population was not contingent on the persistent presence of inducing bacteria. We reasoned that an endogenous self-sustaining autocrine loop was responsible for maintaining cellular stemness. Sterile conditioned media (CM) from infected cells could upregulate SC markers as well as pNF-*κ*B and *β*-catenin in non-infected cells in a dose-dependent manner (Figures 4f(i–ii)), demonstrating that soluble factors secreted by the bacteria-induced CSC fraction can exogenously activate stemness-fostering signalling in surrounding cells.

### Activation of Nod1/Rip2 pathway with simultaneous repression of TLR/MyD88 pathway is responsible for acquisition of stemness properties

Toll-like (TLR) and Nod receptors are involved in microbe recognition and triggering of inflammatory response. Single SK3842 infection caused induction of TLRs 4 and 9 but a drastic reduction of the central TLR adaptor protein, MyD88 (Figure 5a). Expression of cytosolic Nod1 showed no change (Nod2 was undetectable). However, after multiple infections, TLR4 and TLR9 levels showed no change, MyD88 was diminished even further and Nod1 and its main adaptor protein, Rip2, were elevated significantly. The other TLR adaptor protein Trif3 remained unaffected. Since NF-*κ*B was not activated after a single infection (Supplementary Figure S1), it showed that Nod1-Rip2 pathway, not TLR-MyD88 or TLR-Trif3 pathways, is responsible for NF-*κ*B activation. Nod1 knockdown caused attenuation of not only Nod1 but also Rip2 in both control and infected cells (Figure 5b). Ectopic MyD88 expression caused a reciprocal reduction in both Nod1 and Rip2 expression in infected, but not control, cells. Nod1 ablation diminished pNF-*κ*B level down to basal level in infected cells, without affecting it majorly in control cells (Figure 5c). Forced MyD88 expression also resulted in downregulation of pNF-*κ*B in infected cells, unlike in control cells. Nod1 knockdown also caused attenuation of *β*-catenin in infected cells. Most importantly, both Nod1 knockdown and forced MyD88 expression caused a radical reduction in CD44 and CD133 levels in infected cells, resulting in a loss of both enriched CD44^high^ population (Figure 5d) and spheroidogenic ability (Supplementary Figure S4). Thus, simultaneous Nod1/Rip2 activation and TLR/MyD88 repression is instrumental in expansion of CSC population in infected cells.

### Microbe-driven induction of cellular stemness is specific for intestinal epithelial cells and non-pathogenic bacteria

To check whether SK3842 can induce stemness in non-intestinal cell lines and whether another naturally invasive bacteria can recapitulate this phenomenon, we used liver (HepG2) and alveolar (A549) cell lines and wild-type *Shigella flexneri* (M90T). SK3842 did not infect A549 cells but invaded HepG2 cells, although at a much lower efficiency (Supplementary Figure S5A). Both HepG2/SK3842 and Caco-2/*Shigella* cells had highly elevated pNF-*κ*B while *β*-catenin was high in infected HepG2 cells (Supplementary Figure S5B). MyD88 and Nod1 expression was increased in both cells (Figure 5e). However, Rip2 remained unchanged in HepG2 but attenuated significantly in Caco-2/*Shigella* cells, showing that MyD88 signalling was not attenuated and/or Nod1/Rip2 axis was not activated in both cases. No significant changes in CD44 and CD133 levels was seen in either of these cells (Figure 5f), establishing the role of Nod1/Rip2 signalling in inducing stemness. SK3842 infection raised Nod1 and repressed MyD88 levels in differentiated Caco-2 but did not have any significant effect on Nod1 and MyD88 in SC-rich CD44^high^ cells from infected culture (Figure 5g), further confirming that only non-stem cells are responsive to SK3842 invasion.

### Internalized SK3842 can also aggravate tumorigenicity in some aggressive adenocarcinoma cell lines

**Figure 6:**
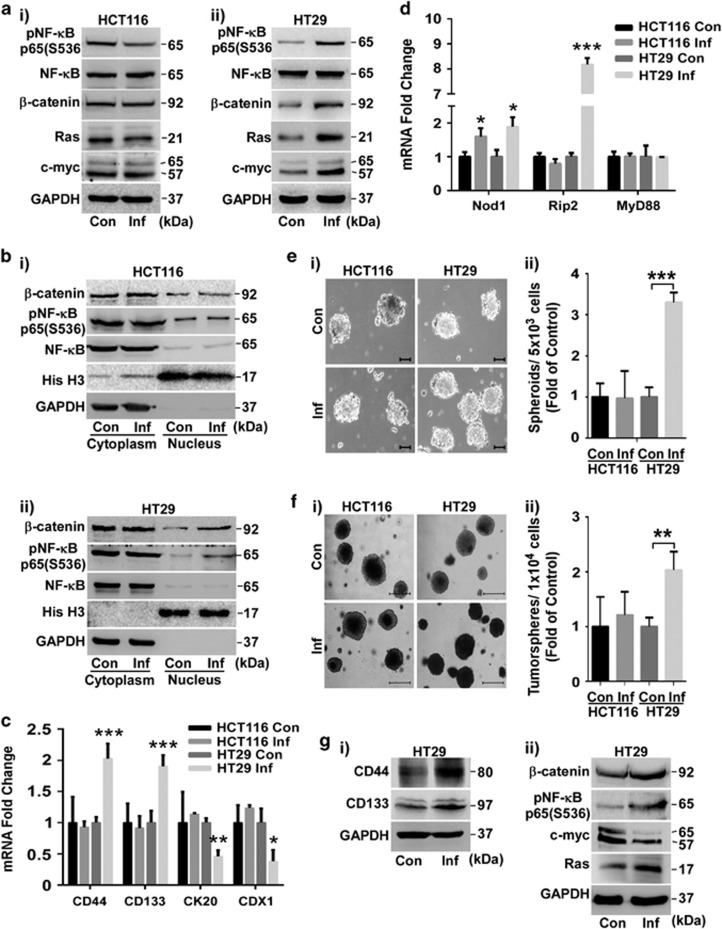
Internalized bacteria induces tumorigenic stemness in aggressive CRC cell lines. (**a**–**f**) Effects of SK3842 infection on HT29 and HCT116 cells. (**a**) Protein levels of pNF-*κ*B, *β*-catenin, Ras and c-myc. (**b**) Cytoplasmic and nuclear levels of NF-*κ*B and *β*-catenin. (**c**) mRNA levels of SC markers CD44 and CD133 and differentiation markers CDX1 and CK20. (**d**) mRNA levels of Nod1, Rip2 and MyD88. (**e**–**f**) Spheroidogenic and tumorigenic ability. (**e**) Spheroid formation and (**f**) Soft agar tumorsphere formation by control and infected cells, showing (**e**(i) and **f**(i)) morphology and (**e**(ii) and **f**(ii)) fold change in numbers. Scale bar=100 *μ*m (**e**(i)) and 500 *μ*m (**f**(i)). (**g**) Expression of indicated proteins related to (i) stemness and (ii) oncogenicity in HT29 tumorspheres. Fold change=Mean±S.D. (**c** and **d**) and Mean±S.E.M. (**e**(ii) and **f**(ii)) for three experiments; *P<0.05, **P<0.01 and ***P<0.001 *versus* control

HCT116 and HT29 are highly aggressive carcinoma cell lines with a very large and an intermediate CSC population, respectively. SK3842 infection increased Mcl1 and reduced Bim and Puma in HT29. It did not affect Mcl1 or Bim but increased Puma in HCT116 cells (Supplementary Figure S6A). Also, infection increased expression (Figure 6a,Supplementary Figure S6B) and nuclear accumulation (Figure 6b) of pNF-*κ*B and *β*-catenin only in HT29 cells. Increase in dedifferentiation markers CD44 and CD133 and repression of differentiation markers CDX1 and CK20 was also specific to HT29 (Figure 6c,Supplementary Figure S6C). Nod1 was upregulated but MyD88 was unaffected in both cell lines (Figure 6d). However, only HT29 showed an increase in Rip2 expression, showing that Nod1-Rip2 axis was responsive in HT29 but not in HCT116. Expansion of CSC fraction in HT29 cells was demonstrated by its enhanced spheroidogenic (Figure 6e) and tumorigenic ability (Figure 6f), without significant changes in the tumorsphere size from control cells. Ras and c-myc levels were increased in infected HT29 cells (Figure 6a; Supplementary Figure S6B). Ras, CD44 and CD133 remained elevated but c-myc showed attenuation in HT29 tumorspheres (Figure 6g). Thus, SK3842 can also aggrandize tumorigenicity in some aggressive adenocarcinoma cells lines with enriched CSC sub-fraction through Nod1/Rip2 axis stimulation.

### SK3842-treated intestinal cells show enhanced tumorigenicity in murine xenograft model

**Figure 7:**
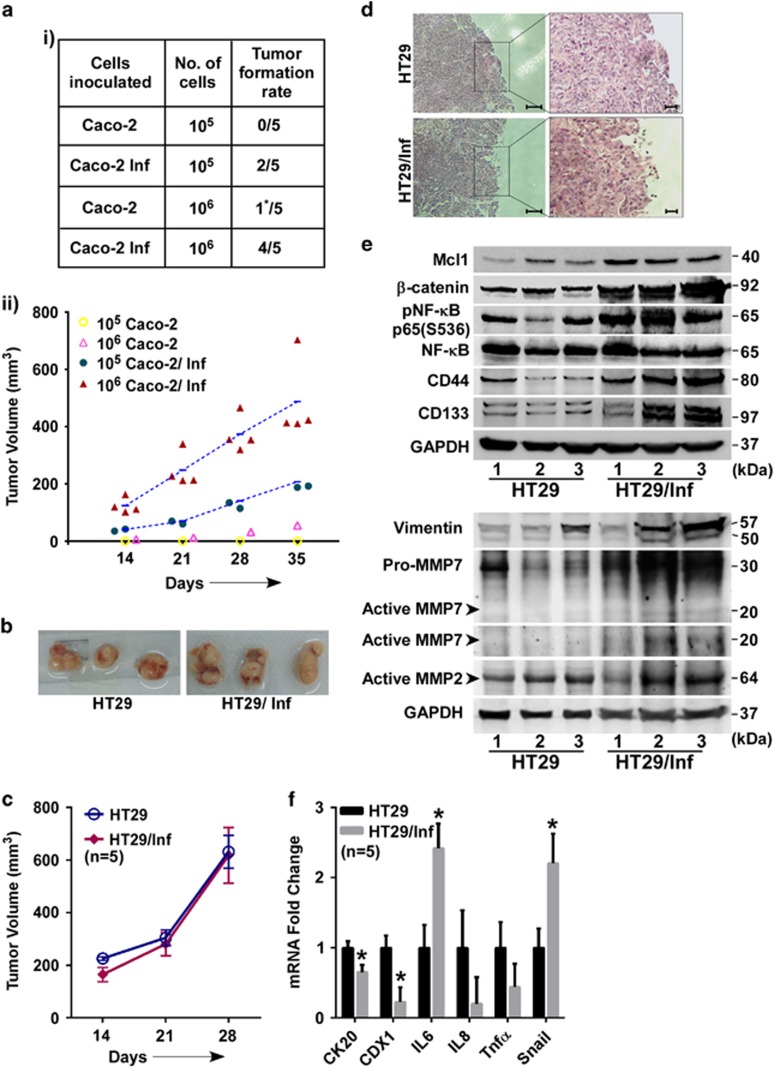
SK3842-infected cells exhibit higher tumorigenicity *in vivo.* (**a**) Effect of SK3842 infection on tumorigenic ability of Caco-2 cells in nude mice: (i) Xenograft tumour formation in different experimental groups at 35 days; (ii) Growth curve of xenografts of control and infected cells. (**b**–**f**) Tumorigenic ability of infected HT29 cells. (**b**) Representative pictures of excised xenografts in nude mice at 28 days (1 × 10^6^ cells). (**c**) Growth curve of xenografts of control and infected cells. (**d**) Haematoxylin and eosin staining of xenograft sections. (**e**) Cellular levels of indicated proteins related to signal transduction, stemness and tumour invasion. (**f**) mRNA levels of cytokines, differentiation markers and EMT transcription factor. Data=Mean±S.E.M. (**c** and **f**); *P<0.05 *versus* control (*n*=5)

Caco-2 cells failed to develop tumours in nude mice at 35 days, except for one barely palpable graft (10^6^ group), a feature observed by others.^18^ However, SK3842-infected Caco-2 yielded robust tumour grafts at 40% frequency in 10^5^ group and 80% frequency in10^6^ group (Figure 7a(i)), validating their vastly advanced tumorigenic capacity. 10^6^ group developed tumours faster and had larger final tumour volumes than 10^5^ set (Figure 7a(ii)). In HT29 cells, there was no significant difference, in either tumour size (Figure 7b), volume or development time (Figure 7c) between control and infected groups, a feature also seen with genotoxin-positive *E. coli*.^6^ However, histological staining revealed that infected group tumours had irregular borders with a large proportion of elongated, less cohesively packed cells (Figure 7d), a common feature of EMT related to tumour metastasis.^19^ Apart from elevated CD44, CD133, Mcl1, *β*-catenin and pNF-*κ*B, these tumours also had high matrix metalloproteinases MMP2 and MMP7and Vimentin which are strong correlates of tissue invasion and metastasis (Figure 7e). Dedifferentiation markers, CK20 and CDX1, and inflammatory cytokine IL6 were also elevated in these tumours as was the EMT transcription factor Snail (Figure 7f). Collectively, tumour cells from infected HT29 displayed features of poorer differentiation, enhanced inflammation and greater migration.

## Discussion

Commensal intestinal *E. coli* with virulence traits are linked to malignant transformation of IECs upon disruption of the intestinal barrier.^20^ We show that long-term internalization of non-pathogenic *E. coli* can also instigate neoplastic progression in IECs through pro-survival and self-renewing traits rather than through genomic damage, thereby linking dysbiotic behaviour of a benign gut commensal to CRC oncogenesis. Unlike most studies which use genetically engineered animals, we used an engineered constitutively invasive form of *E. coli* to recapitulate a breach of traditional host-commensal compartmentalization. Aberrant bacterial invasion led to subversion of host signal transduction functions through reciprocal modulation of microbe-sensing PRRs (Nod1/Rip2 activation and MyD88 repression), leading to mutually re-enforcing activation of the central pathways which control cellular differentiation and oncogenic status – NF-*κ*B and *β*-catenin.^21, 22^ Modulations of host cell signalling machinery resulted in the acquisition of augmented malignancy traits related to apoptosis resistance, mobility, self-renewal and tumorigenicity, orchestrated by changes in expression of effectors with proven roles in tumorigenicity. Tumour-promoting cytokines IL6 and Tnf*α*,^23^ multipotency transcription factors Sox2, Oct4 and Nanog^24^ and oncogenic factors c-myc and Ras^25^ were all upregulated in invaded host cells. Over-expression of multipotency factors^26^ and combined activation of oncogenic Ras and c-myc^27^ can reprogram colon cells to CSCs. Like most induced SCs, microbe-induced CSCs did not require the inducing agent for maintenance of undifferentiated state^28^ and could induce stemness in non-infected cells through secreted factors. SK3842 markedly enhanced the tumorigenicity of poorly tumorigenic Caco-2 and amplified stemness, inflammation and migration traits in aggressive carcinoma cell line HT29. Non-transformed cell line HIEC-6 (data not shown) and highly undifferentiated carcinoma cell line HCT116 were refractory, probably indicating a requisition for predisposing genetic background (dysregulated *β*-catenin and/or non-functional p53, respectively) for CSC expansion. Also, HCT116 is phenotypically and genetically quite distinct from the other two cell lines (wild-type adenomatous polyposis coli (APC), mismatch repair deficiency (MMR^−^), stochastic but not CSC mode of cell maintenance^29^). Further work might shed more light on the roles of p53, APC and MMR in cell fate decisions related to stemness in CRC cells. Other pathogenic bacteria have been linked to dedifferentiation^30^ or oncogenic transformation.^31, 32^ Reprogramming host cell to self-renewing stem-like state has been shown to be a survival and dissemination strategy for bacteria without sophisticated virulence arsenal.^30^ Cellular transition from nonstem state to stemness was restricted to differentiated IECs since intestinal SCs and non-intestinal cells were immune to SK3842-mediated effects. Although our experimental results point to a NF-*κ*B and *β*-catenin activation-mediated dedifferentiation of committed IECs to CSCs, we cannot unequivocally exclude the possibility of selective stabilization and expansion of resident CSC population in infected cultures through autocrine and paracrine signalling by activated cytokines and growth factors. Further experimental work like lineage tracing can distinguish between these two possibilities in future.

CSCs are a select subpopulation of tumour cells that drives tumour initiation, progression and metastasis.^33^ Aberrant activation of *β*-catenin signalling is a ‘gateway' event in CRC oncogenesis that leads to expansion of the tumour-initiating CSCs in the intestine.^34^ Concerted activation of NF-*κ*B and *β*-catenin has been linked to dedifferentiation of lineage-committed epithelial cells to tumour-initiating CSCs,^35^ which is consistent with the established links between inflammation and cancer.^36^ Mutation-induced *β*-catenin activation is not sufficient for CSC phenotype without cooperative mutations (KRAS) or microenvironmental signals (ROS). Our model proposes that, in cells with an existing ‘first hit' *β*-catenin dysregulation, chronic intracellular residence of non-virulent microbes can act as the complementary event to promote tumour-inducing stemness traits. Defective pathogen recognition and aberrant inflammatory signalling leads to chronic inflammation and tumorigenesis.^37^ Pathogenic commensals have highly evolved machinery to hijack or evade immune surveillance that are lacking in bacteria without virulence factors. But, for inflammation-mediated amplification of CSCs to be promulgated by non-pathogenic microbes, constitutive NF-*κ*B activation has to be balanced against uncontrolled inflammation which can lead to both tissue injury and elimination of the instigating microbe. TLR-MyD88 stimulation by commensal bacteria is needed for host-commensal homeostasis and prevention of gut injury.^38^ Host-commensal spatial separation is completely breached in MyD88^−/−^ cells^39^ such that luminal microbes are in direct contact with IECs. Although TLR/MyD88 activity is associated with tumour promotion and Nod activation with tumour prevention,^40^ our study shows that depending on the nature and cellular location of the specific commensal, the roles can be reversed. Microbe-mediated suppression of TLR/MyD88 signalling with concomitant activation of Nod1/Rip2 can subvert self-recognition of commensals, rupture the colonic barrier and induce controlled NF-*κ*B activation concurrently. Our finding that combined stimulation of MyD88 and Nod1, over-activation of NF-*κ*B and pathogenic *Shigella* were all ineffective as inducers of CSC amplification is consistent with the concept of moderately elevated NF-*κ*B levels designating chronic inflammation^41^ state for expansion of CSCs by aberrant behaviour of non-virulent *E. coli*.

Our study shows that oncogenic behaviour of commensals may not always align with the circumscribed parameters of genetically defined pathogenesis. Microbe-driven oncogenicity is an outcome of self-derived and contextual cues determining the role of the microbe in host cell fate decision regarding homeostasis and tumorigenesis.

## Materials and Methods

### Cell lines, bacterial strains and plasmids

Human colorectal adenocarcinoma cell lines Caco-2, HT29 and HCT116 and human hepatocellular carcinoma cell line HepG2 were purchased from ATCC, Manassas, VA, USA. Bacterial strains (SK3842, O157:H7 strain EDL933 and *Shigella flexneri* M90T) were cultured as described previously.^14^Cells were not passaged from the start of the experiment (first infection) to the final cell collection. Plasmid pCMV-HA-MyD88 was purchased from Addgene, Cambridge, MA, USA and shRNA plasmid constructs for Nod1 and I*κ*B*α* were purchased from Santa Cruz Biotech, Dallas, TX, USA. Plasmid phly287 was constructed by amplifying the *hlyE* gene from MG1655 strain using 5′-TCGTTCTCTTATCTTGTGCTC-3′ and 5′-TTCGTAGCCCTGTTTTTGAAAG-3′ as forward and reverse primer, respectively. The amplified fragment was cloned at the *Sma*1 site of puc19 plasmid. EDL933 (HU*α*E38K,V42L) was constructed by P1 transduction and selection of P1-transductants on spectinomycin-agar plates.

### Chemical inhibitors and antibodies

Chemical inhibitors Bay11-7082 (1 *μ*M), JW67 (25 *μ*M) and Lithium chloride (40 mM) were purchased from Sigma Aldrich, St. Louis, MO, USA.

All primary antibodies were purchased from Cell Signalling Technology, Danvers, MA, USA unless otherwise mentioned. Antibodies used were Mcl1 (Cat. No. – 4572), Bim (Cat. No. – 2819), Puma (Cat. No. – 4976), Caspase 3 (Cat. No. – 9662), Caspase 9 (Cat. No. – 9502), *β*-catenin (Cat. No. – 9582), Phosphor-NF-*κ*B (S536) (Cat. No. -3033), NF-*κ*B (Cat. No. – 8242), Phospho-ERK p42/44 (T202/Y204) (Cat. No. – 4370), ERK p42/44 (Cat. No. – 4695), Phospho-p38 MAPK (T180/Y182) (Cat. No. – 4511), Phospho-SAPK/JNK (T183/Y185) (Cat. No. – 4668), Phospho-MEK1/2 (S217/221) (Cat. No. – 9154), Phospho-c-Raf (S338) (Cat. No. – 9427), Phospho-I*κ*B*α* (S32) (Cat. No. – 2859), I*κ*B*α* (Cat. No. – 4814), Ikkα (Cat. No. – 2682), Ikkβ (Cat. No. – 2678), Phospho-Akt (S473) (Cat. No. – 4060), Akt (pan) (Cat. No. – 4691), Phospho-PTEN (S380) (Cat. No. – 9551), Ras (Cat. No. – 3339), c-Myc (Cat. No. – 5605), Cyclin-D1 (Cat. No. – 2926), Phospho-GSK3β (S9) (Cat. No. – 9323), Bax (Cat. No. – 2772), Bcl-2 (Cat. No. – 2870), Bcl_xL_ (Cat. No. – 2764), Survivin (Cat. No. – 2808), c-IAP1 (Cat. No. – 4952), XIAP (Cat. No. – 2045), RhoA (Cat. No. – 2117), Rac1/2/3 (Cat. No. – 2465), Cdc42 (Cat. No. – 2466), CD44 (Cat. No. – 3570), CD133 (Cat. No. – A0219; Neo Scientific, Cambridge, MA, USA), PARP (Cat. No. – sc-53643; Santa Cruz Biotechnology, Dallas, TX, USA), Vimentin (Cat. No. – 3932), E-cadherin (Cat. No. – 3195), MMP-2 (Cat. No. – 4022), MMP-7 (Cat. No. – sc-80205; Santa Cruz Biotechnology, USA), Histone H3 (Cat. No. – 4499) and GAPDH (Cat. No. – 5174). Alexa Fluor 488- Phalloidin (Cat. No. – A12379) and FITC-CD44 (Cat. No. – MHCD4401) were purchased from Invitrogen, Carlsbad, CA, USA. HRP-conjugated goat anti-mouse IgG (H+L) secondary antibody (Cat #: 62-6520) and HRP-conjugated goat anti-rabbit IgG (H+L) secondary antibody (Cat #: 65-6120) from Thermo Fisher Scientific (Waltham, MA, USA) were used as secondary antibodies.

### Cell culture and bacterial infection

Caco-2, HT29, HCT116 and HepG2 cells were grown in Dulbecco's modified Eagle's media (DMEM, Gibco, Waltham, MA, USA) supplemented with 10% heat inactivated fetal bovine serum (FBS) (Gibco, USA), 2 mM Glutamine (Gibco, USA) and 100 *μ*g/ml gentamicin (Corning, New York, USA) in a humidified incubator at 37 °C with 5% CO_2_. Caco-2 cells were subcultured after reaching 70–80% confluency. For Caco-2 differentiation, cells were grown on Corning BioCoat HTS 1.0 *μ*m filter support transwell plates in basal seeding medium for 2 days and Entero-STIM Enterocyte Differentiation Medium (EDM) for 1 day, according to the manufacturer's instructions (Corning, New York, USA).

Infection with SK3842 was optimized to three infections over a period of 10 days (first infection 24 h post seeding and remaining two infections every fourth day). Cells were collected 24 h after the last infection on the 11th day. Infection time was 3 h for SK3842, EDL933 and *Shigella* in gentamicin-free DMEM which was replaced with fresh gentamicin-DMEM until next infection. For pathway inhibition studies, inhibitors were maintained throughout the infection duration. For clearance of SK3842, cells were cultured in DMEM with 100 *μ*g kanamycin (Kan) for 4 days after final infection. Kan treated cells were checked for the presence of SK3842 by lysing the cells in mammalian cell lysis buffer (Sigma Aldrich, USA) and plating the lysates on LB-spectinomycin (25 *μ*g) agar plates.

### Microscopy

Bright field images were captured from at least three random fields at × 4 magnification (for soft agar tumour spheres) and × 10 magnification (for adherent cultures and spheroids) in TS100 eclipse inverted microscope (Nikon, Minato, Tokyo, Japan).

For immunofluorescence microscopy, cells were labelled with Alexa 488-Phalloidin or FITC-CD44 (Thermo Fisher Scientific, USA) following manufacturer's protocol and mounted with anti-fade mounting medium containing DAPI (Thermo Scientific, USA). Fluorescent images were acquired with × 100 objective lens using Axiovert 200 inverted fluorescence microscope (Carl Zeiss, Oberkochen, Germany) and multi-dimension acquisition module of AxioVision Rel. software v4.8.2. Images were captured using identical settings and acquisition time.

### Serum starvation and anoikis resistance

For serum starvation, cells were cultured in serum-free DMEM for 96 h. For anoikis resistance, cells were seeded in six-well plates pre-treated with poly-HEMA and cultured for 96 h. Cell viability was assessed by MTT assay (Sigma-Aldrich, USA).

### Reporter assay for NF-*κ*B and *β*-catenin

For NF-*κ*B reporter assay, cells were co-transfected with a mixture of pGL 4.32 plasmid (Promega, Madison, WI, USA) containing the NF-*κ*B response element linked to a firefly luciferase reporter gene and pGL 4.17 plasmid (Promega, USA) containing Renilla luciferase reporter gene at a ratio of 1 : 50, 24 h before collection of cells. Luciferase activity was assayed using Dual-Luciferase Reporter Assay System (Promega, USA). For the *β*-catenin reporter assay, we used Cignal TCF/LEF reporter assay kit (SA Biosciences, Frederick, MD, USA) which contained the *β*-catenin response element linked to a firefly luciferase reporter gene and pGL 4.17 plasmid containing Renilla luciferase reporter gene at a ratio of 1 : 40. Plasmids were transfected using Lipofectamine reagent (Thermo Scientific, USA) and luciferase activity measured with FLUOstar Omega Multi-Mode Microplate Reader with CCD-based Spectrometer (BMG Labtech GmbH, Ortenberg, Germany).

### Transwell invasion assay

Cell migration was assessed by Transfilter assays using 8.0 *μ*m pore inserts (Corning, USA) in six-well plates. Overnight serum starved cells were seeded onto the upper chamber of the inserts at a density of 5 × 10^5^ cells per well in serum-free DMEM and 1 ml 10% FBS-DMEM was layered onto the lower chamber to serve as a chemo-attractant. After 18 h, upper surface was wiped with a cotton swab to remove non-invasive cells. Invasive cells adhering to the lower surface of the membrane were fixed with 4% paraformaldehyde and stained with crystal violet (Sigma Aldrich, St. Louis, MO, USA). Percentage of cells that invaded through the transwell filter was calculated and represented as fold of control.

### Spheroid forming assay

Cells were trypsinized and 5 × 10^3^ cells/0.5 ml per well were plated onto agarose coated six-well plates in SFM which consisted of serum-free DMEM with 1% B27 nutrient mixture minus Vitamin A, 20 ng/ml of epidermal growth factor and 10 ng/ml of fibroblast growth factor (all from Invitrogen, USA). Media was replenished every 2 days. Formation of sphere-like structures was visible after 2 days and the photographs of each group were captured after 5 days. Number and area of spheroids were calculated by ImageJ software (NIH, USA), area represented as Arbitrary Units (AU).

### Knockdown of Nod1 and I*κ*B*α* and ectopic expression of MyD88

To stably knock down Nod1 and I*κ*B*α*, cells were transfected with a set of shRNA constructs against Nod1 or I*κ*B*α* (200 ng/10^4^ cells) or scrambled shRNA and then selected with puromycin (0.5 *μ*g/ml) according to the manufacturer's instructions. Knockdown cells were used for further bacterial infection experiments. To ectopically express Myd88, cells were transfected with pCMV-HA-MyD88 plasmid (40 ng/10^4^ cells) 24 h before collection of cells.

### RNA isolation, cDNA synthesis and real-time PCR analysis

Total RNA from cell culture was isolated using TriZol Reagent (Life Technologies, Carlsbad, CA, USA) as per the manufacturer's instructions. RNA was subjected to DNase treatment (TURBO DNA Free Kit, Ambion, Carlsbad, CA, USA) to eliminate genomic DNA contamination. For cDNA synthesis, 2 *μ*g of quantified DNase treated RNA was reverse transcribed with reverse transcriptase, SuperScript II RT, (Invitrogen USA) using random hexamer (Applied Biosystems, Foster City, CA, USA). For total RNA isolation, approximately 100 mg of excised xenograft tumours stored in RNAlater (Ambion, Carlsbad, CA, USA) were homogenized in TriZol Reagent using Tissue Grinder (Genetix, Biotech Asia, New Delhi, India) in addition to the protocol followed for cultured cells.

Primers for qPCR were designed using Primer3 software or referred from literature (Table 1). qPCR was performed using SYBR Green Chemistry (KAPA SYBR FAST Master Mix 2 ×, KAPABIOSYSTEMS, Wilmington, MA, USA) in LightCycler 480 (Roche, Mannheim, Germany) in 96-well format with the following cycling conditions: Initial denaturation 95 °C–2 mins; amplification cycle: denaturation 95 °C–15 s, annealing 60 °C–20 s and extension 72 °C–5 s. Amplicon specificity was confirmed by melting curve analysis.

Expression of marker transcripts was quantified by qPCR using GAPDH and 18S rRNA as housekeeping controls. The differential fold change in the expression was calculated by 2^(−ΔΔCt)^ method.

End-point PCR targeting the 16S rRNA gene was used to check for the presence of SK3842 in 10 days adherent and SK3842 infected CD44^high^ and CD44^low^ cells. Human GAPDH was used as loading control. Primers used for 16S rRNA were: 5′-ACAATGGGCGCAAGCCTGATG-3′ (Forward) and 5′-CGGTCGACTTAACGCGTTAGC-3′ (Reverse).

### Protein isolation and western blotting

Protein isolation and western blotting procedures have been described before.^14^ Whole cell lysates from cell cultures were prepared by adding lysis buffer (20 mM Tris-HCl, pH 7.5; 150 mM NaCl; 1 mM EDTA, pH 8; 0.1% NP40; 0.01% SDS with protease inhibitor cocktail and phos-STOP (Roche, Germany).

For xenograft tumour lysate preparation, excised, snap frozen tumours were homogenized in above mentioned lysis buffer using Tissue Grinder, sonicated (50% amplitude for 2 min with 10 s on and 20 s off pulses) and lysates were cleared by centrifugation.

Total proteins isolated were quantified by Bradford assay (Bio-Rad, Hercules, CA, USA) and equal amounts of lysates were resolved in appropriate SDS-PAGE gels, transferred onto 0.45 *μ*m PVDF membrane (Millipore, Banglore, India) and probed with primary antibodies. HRP-conjugated secondary antibodies and Supersignal West Pico chemiluminescent substrate (Pierce, Rockford, IL, USA) were used for protein band detection using GeneGnome system (Syngene, Cambridge, UK). Anti-GAPDH alone or in combination with anti-histone-H3 antibody was used as protein loading and transfer control.

#### Cell fractionation

Nuclear and cytoplasmic fractions were isolated using NE-PER Nuclear and Cytoplasmic Extraction Reagents (Pierce, USA) as per the manufacturer's instructions. Protein quantification and western blotting was done as described above. GAPDH and Histone H3 antibodies were used to validate the cytoplasmic and nuclear fractions respectively.

### Conditioned media experiment

Conditioned media from uninfected and SK3842 infected Caco-2 cells was collected and made free from dead cells and debris by filtration through 0.22 *μ*m syringe filter (Millipore, India). Pre-seeded Caco-2 cells were then incubated in 30 and 70% CM in 10% FBS containing DMEM, from both control and infected Caco-2 cells for 24 h. These cells were used for further analysis.

### Flow cytometry

Cells were collected by gentle scraping, washed (2 ×) and suspended in PBS with 2% FBS. 1 × 10^6^ cells were stained with FITC-CD44 for 30 min at room temperature. After two washes, cells were finally resuspended in 0.5 ml sheath fluid for flow cytometric analysis. Cytometry was performed using BD LSR II (Becton Dickinson, Franklin Lakes, NJ, USA) and BD FACSDiva Software v6.1.3 was used for data analysis. Unstained Caco-2 cells were used as controls for setting up the cell size gate to exclude cell debris and clumps.

#### Fractionation of CSC and non-CSC fractions

Cells were stained with FITC-CD44 antibody for 30 mins and sorted by FACSAria II cell sorter. CD44^high^ CSCs and CD44^low^ non-CSCs populations were sorted again to increase their purity (>99.2% in all cases). CSCs were cultured in spheroid-forming medium. Non-CSCs were cultured overnight in DMEM plus 5% FBS and were transferred to SFM next day. For checking SK3842 re-infection of CD44^high^ and CD44^low^ fractions of infected cells, bacteria were treated with kan 24 h after the third infection to remove the internalized bacteria before cell sorting. After cell sorting, the cells in spheroid medium were exposed to SK3842 at a MOI of 1 : 100 for 3 h and checked for the presence of bacterial 16S rRNA DNA or Nod1 and MyD88 mRNA after 12 h. For soft agar assay, cells were acclimatized in SFM for 24 h after sorting and then plated for tumorsphere formation.

### Soft agar colony formation assay

Cells were trypsinized and 1 × 10^4^ cells/well were suspended in DMEM containing 20% FBS, 4 mM glutamine and 200 *μ*g/ml gentamicin with 0.35% agarose and seeded in six-well plates pre-coated with 1% agarose in 5% FBS containing DMEM. Media (200 *μ*l) was replenished every 2 days. Cells were allowed to form colonies for 21 (or 14) days. Number and area of tumorspheres were calculated using ImageJ software (NIH, USA) and area represented as arbitrary units (AU).

### Xenograft experiment

All animal experiments were performed in accordance with the Institutional Animal Care and Use Committee procedures and guidelines of the Institute of Liver and Biliary Sciences, India. For xenograft experiment, 5-week-old male athymic nude mice (strain NCR/nu/nu) were obtained from Vivo Bio Tech Ltd (Hyderabad, India) and randomly separated into different groups. Mice were acclimatized for 7 days post arrival. They were housed in sterile filter-topped cages with *ad libitum* access to food and water at 21±2 °C with a 12 h light/dark cycle. 10 dpi Caco-2 and HT29 cells from control and infected groups were collected by trypsinization and approximately 1 × 10^5^ cells (Caco-2) and 1 × 10^6^ cells (Caco-2 and HT29) in 0.2 ml PBS were implanted subcutaneously at the back of the neck of each animal. Mice were maintained in sterile conditions and monitored for body weight and tumour growth. Tumour size measurements were initiated 14 days post injection and monitored every 5 days for 28 and 35 days for HT29 and Caco-2 groups, respectively. Tumour diameters (length and width) were measured using digital Vernier callipers and tumour volume in mm^3^ was calculated as length × (width)^2^/2. After 4 and 5 weeks (for HT29 and Caco-2 cells, respectively), mice were killed and tumour tissues were collected for further analysis.

### Statistical analysis

All statistical analyses were performed using GraphPad Prism6 software. Two-tailed unpaired *t*-test was utilized for comparison of two groups. Values were considered significantly different at *P*<0.05 and expressed as Mean±S.D. or Mean±S.E.M.

## Figures and Tables

**Table 1:** Primer sets used for quantitative real-time PCR

**Gene**	**Forward (5′-3′)**	**Reverse (5′-3′)**
IL6	CCTTCCAAAGATGGCTGAAA	CAGGGGTGGTTATTGCATCT
IL8	ATGACTTCCAAGCTGGCCGTGGCT	TCTCAGCCCTCTTCAAAAACTTCTC
Tnf*α*	CCCAGGGACCTCTCTCTAATC	ATGGGCTACAGGCTTGTCACT
Snail	ACCACTATGCCGCGCTCTT	GGTCGTAGGGCTGCTGGAA
Slug	TGTTGCAGTGAGGGCAAGAA	GACCCTGGTTGCTTCAAGGA
CD44	CCGCTATGTCCAGAAAGGA	CTGTCTGTGCTGTCGGTGAT
CD133	ACGCACGTAGGGAATGGAT	GGTTTGCACGATGCCACTTT
c-myc	TCAAGAGGCGAACACACAAC	GGCCTTTTCATTGTTTTCCA
Sox2	AAGGGTTCTTGCTGGGTTTT	AGACCACGAAAACGGTCTTG
Lgr5	CTCTTCCTCAAACCGTCTGC	GATCGGAGGCTAAGCAACTG
Oct4	GTTGATCCTCGGACCTGGCTA	GGTTGCCTCTCACTCGGTTCT
Nanog	GTCTTCTGCTGAGATGCCTCACA	CTTCTGCGTCACACCATTGCTAT
TLR4	CAGAGTTGCTTTCAATGGCATC	AGACTGTAATCAAGAACCTGGAGG
TLR9	TGGTGTTGAAGGACAGTTCTCTC	CACTCGGAGGTTTCCCAGC
MyD88	GACCCCTGGTGCAAGTACC	AGTAGCTTACAACGCATGACAG
Trif3	ACGCCACTCCAACTTTCTGT	TCAGGTGAGCTGAACAAGGA
Nod1	TCCAAAGCCAAACAGAAACTC	CAGCATCCAGATGAACGTG
Rip2	CCATCCCGTACCACAAGCTC	GCAGGATGCGGAATCTCAAT
CK20	CAGACACACGGTGAACTATGG	GATCAGCTTCCACTGTTAGACG
CDX1	TCGGACCAAGGACAAGTACC	TGTTGCTGCTGCTGTTTCTT
GAPDH	GGTTTCTATAAATTGAGCCCGCA	ACCAAATCCGTTGACTCCGA
18S rRNA	GGAGTATGGTTGCAAAGCTGA	ATCTGTCAATCCTGTCCGTGT
