# Supplementary material for: Induction of intestinal stemness and tumorigenicity by aberrant internalization of commensal non-pathogenic E. coli
Source: Cell Death Dis. 2017 Mar 16;8(3):e2667–. doi: 10.1038/cddis.2017.27 (PMC5386548; doi:10.1038/cddis.2017.27)
Supplement: Supplementary Information [file cddis201727x1.pdf]

Induction of intestinal stemness and tumorigenicity by aberrant internalization of commensal non-pathogenic *E.coli*

Sahu et al.

Supplemental Information

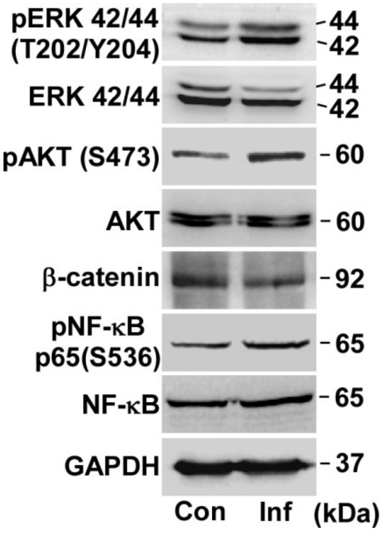

Fig. S1: Major signalling pathways are not altered significantly following 1 round of SK3842 infection. Western blot analysis of ERK, AKT, β-catenin and NF-κB proteins in control and SK3842-infected Caco-2 cells following 1 infection**.** Cells were collected 24 hrs after a single SK3842 infection.

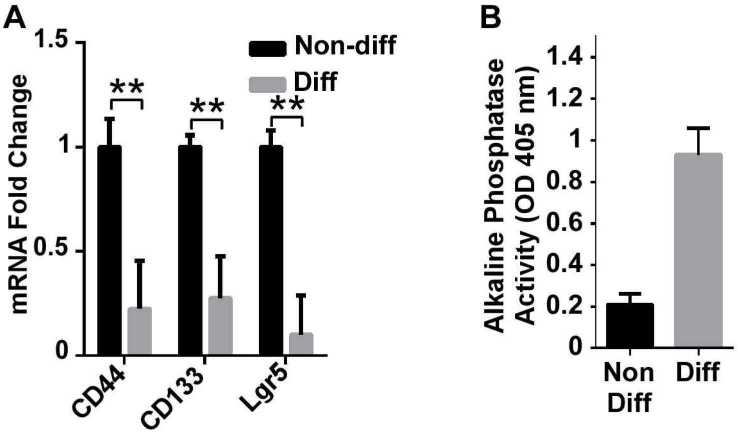

Fig. S2: Differentiation of Caco-2 cells. Sub-confluent Caco-2 cells were differentiated by Corning BioCoat HTS Caco-2 Assay system (see Materials and Methods section). Differentiation was

confirmed by **(A)** mRNA expression of CSC markers CD44 and CD133 and ISC marker Lgr5 and **(B)** Alkaline phosphatase activity in non-differentiated and differentiated cells. Data = Mean ± SD (Three experiments); ** P< 0.01 versus non-differentiated control.

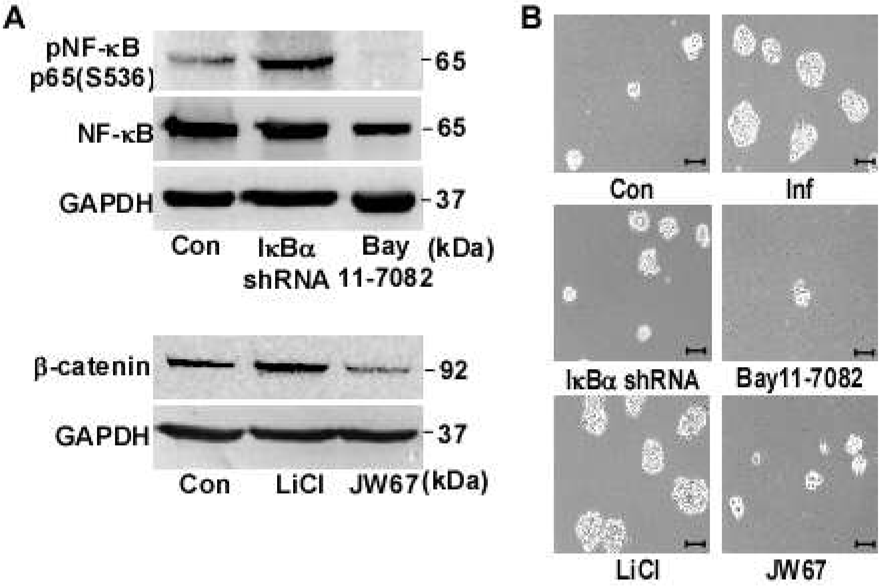

Fig. S3: Spheroid forming ability is affected in SK3842-infected Caco-2 cells with altered NF-κB and β-catenin expression. (A) Efficacy of pathway manipulations on the respective proteins following gene knockdown or inhibitor treatment. Over-expression and repression of NF-κB pathway was done by using IκBα shRNA (to knock down NF-κB inhibitor IκBα) and Bay11-7082 (inhibitor of IκBα negative phosphorylation), respectively. For β-catenin pathway, LiCl (inhibitor of GSK3β) and JW67 (inducer of β-catenin destruction) were used for activation and suppression, respectively. Status of NF-κB and β-catenin protein levels was checked prior to SK3842 infection **(B)** Spheroidogenic ability of infected cells with altered NF-κB and β-catenin background. Cells were collected 24 hrs after the 3rd infection and plated in spheroid forming media for 5 days. Scale bar= 100 μm.

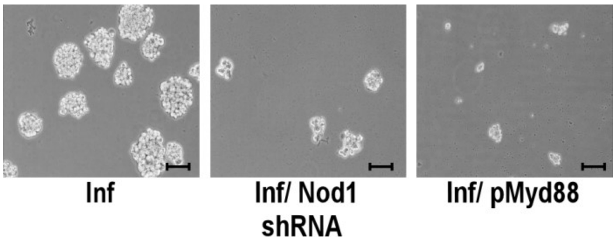

Fig. S4: Nod1 ablation and MyD88 ectopic expression hinders spheroid-forming ability of SK3842-infected cells. Spheroid forming ability of SK3842-infected cells in Nod1-minus (Nod1 shRNA) and ectopically expressing MyD88 (pCMV-HA-MyD88 plasmid) background. Nod1 knockdown was done prior to first infection and MyD88 plasmid was transfected 24 hrs prior to collection of cells. Cells were plated in spheroid-forming media for 5 days. Scale bar= 100 μm.

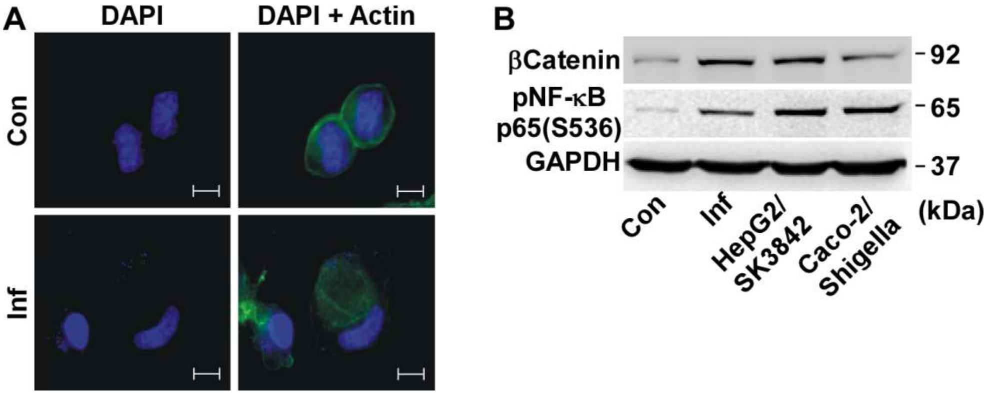

Fig. S5: SK3842 invasion of HepG2 cells and *Shigella* infection of Caco-2 cells. (A) Detection of internalized SK3842 in HepG2 cells after 3rd round of bacterial infection. HepG2 cells were stained with Alexa Fluor 488-Phalloidin and DAPI. Scale bar= 2 μm. **(B)** Protein levels of pNF-κB and β-catenin in SK842-infected HepG2 and *Shigella*-infected Caco-2 cells following 3 rounds of infections.

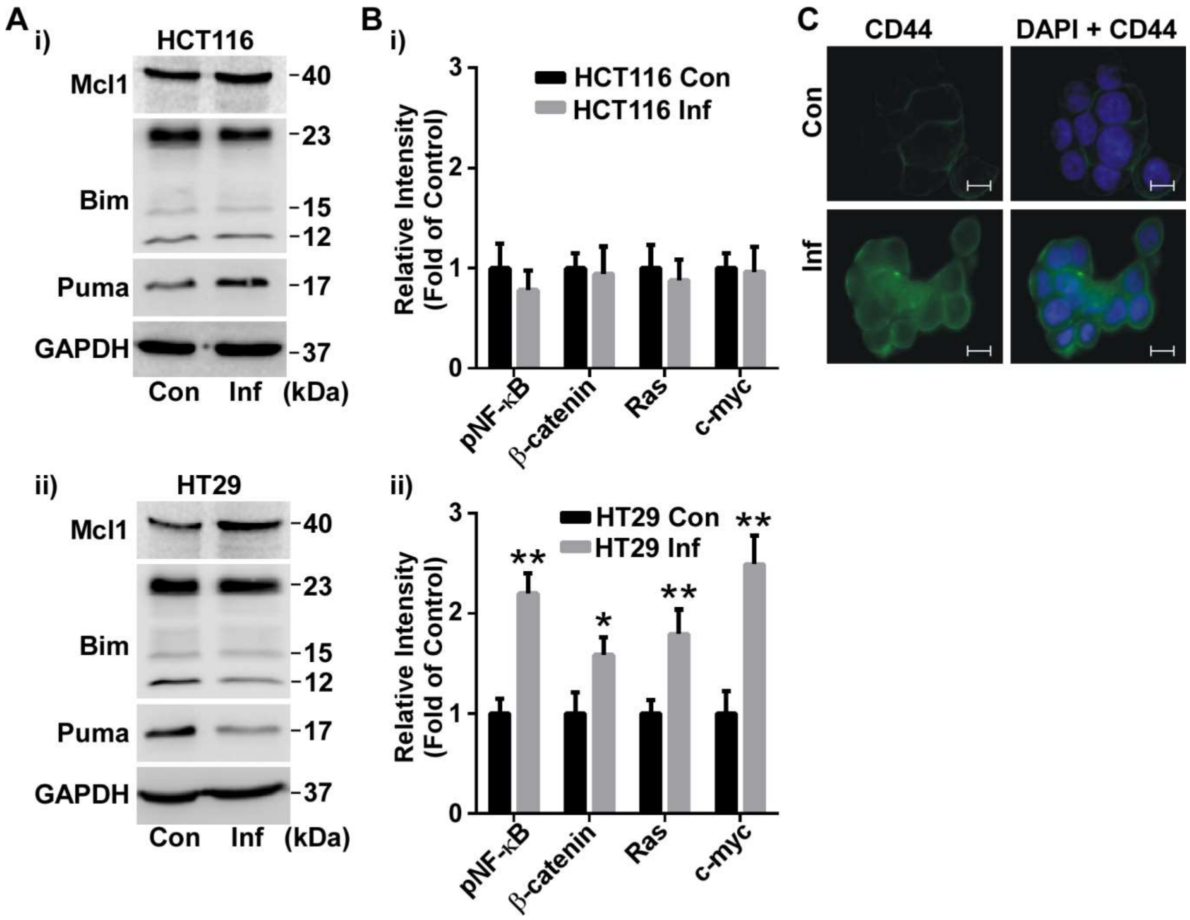

Fig. S6: SK3842 infection of CRC cell lines HCT116 and HT29: (A) Protein levels of Mcl-1, Bim and Puma after 3 infections of SK3842 in **(i)** HCT116 and **(ii)** HT29 cell line **(B)** Summary histogram representing fold changes in levels of indicated proteins (Representative blot shown in Fig. 6A) in **(i)** HCT116 cell line and **(ii)** HT29 cell line **(C)** Immunofluorescent staining of CD44 in HT29 cells using FITC-labelled CD44 antibody and DAPI. Scale bar= 2 μm. Data = Mean ± SD (Three experiments); * P< 0.05 and ** P< 0.01 versus control.
